# Association between visit-to-visit fasting glycemic variability and depression: a retrospective cohort study in a representative Korean population without diabetes

**DOI:** 10.1038/s41598-022-22302-0

**Published:** 2022-11-04

**Authors:** Hye Jun Kim, Sung Min Kim, Gyeongsil Lee, Seulggie Choi, Joung Sik Son, Yun Hwan Oh, Soo Jung Choi, Seogsong Jeong, Sang Min Park

**Affiliations:** 1 Department of Biomedical Sciences, Seoul National University Graduate School, Seoul, Republic of Korea; 2 Department of Family Medicine, Seoul National University Hospital, Seoul, Republic of Korea; 3 Department of Family Medicine, Chung-Ang University Gwangmyeong Hospital, Chung-Ang University College of Medicine, Gwangmyeong-Si, Republic of Korea; 4 Department of Family Medicine, Gachon University Gil Medical Center, Incheon, Republic of Korea; 5 Department of Biomedical Informatics, CHA University School of Medicine, Seongnam, Republic of Korea

**Keywords:** Diseases, Endocrinology, Medical research, Risk factors

## Abstract

Glycemic variability (GV) is a risk factor for depression in patients with diabetes. However, whether it is also a predictor of incident depression in people without diabetes remains unclear. We aimed to investigate the association between visit-to-visit variability in fasting serum glucose (FSG) levels and the incidence of depression among Koreans without diabetes. This retrospective cohort study included data of people without diabetes who did not have depression at baseline and had at least three FSG measurements (n = 264,480) extracted from the 2002–2007 Korean National Health Insurance Service–National Health Screening Cohort. GV was calculated as the average successive variability of FSG. Among 264,480 participants, 198,267 were observed during 2008–2013 and their hazard ratios (HR) of incident depression were calculated. Participants with the highest GV showed a higher risk of depression in fully adjusted models than those with the lowest GV (HR, 1.09; 95% CI, 1.02–1.16). The risk of incident depression heightened with increasing GV (*p* for trend < 0.001). Greater visit-to-visit GV may be associated with the risk of developing depression in people without diabetes. Conversely, maintaining steady FSG levels may reduce the risk of incident depression in people without diabetes.

## Introduction

Glycemic variability (GV) refers to fluctuations in blood glucose levels over a short (within- or between-day variability)- or long-term period (months or years) and is an integral component of glucose homeostasis^1–3^. While HbA1c has long been considered as the gold standard for measuring glycemic control, GV is increasingly gaining its clinical significance as a more meaningful measure of glycemic control compared to HbA1c^2^. Despite the controversial findings, a few studies have demonstrated that GV is associated with adverse clinical outcomes such as cardiovascular events, hypoglycemia, micro- and macrovascular complications, and mortality rates^2,4–6^. Besides, GV is reported to be correlated with psychiatric diseases such as depression and cognitive disorder^2,7–10^. In fact, a certain degree of GV can be observed in all groups, from people without diabetes to those with impaired glycemic levels or diabetes^11,12^. For example, exposure to variability in blood glucose levels was found to be more harmful than an episode of acute or chronic stable hyperglycemia in both normal and people with type 2 diabetes^11,13^. Based on the aim of treating diabetes to restore glycemia to that of persons without diabetes, examining the distribution of GV and its impact should begin among general populations without diabetes^14^. However, to the best of our knowledge, there have been no studies that investigated the impact of GV on the onset of depression in people without diabetes. When glucose fluctuates, excessive activation of oxidative stress and vascular damage are induced, all of which can contribute to developing depression^10,13^. Based on a prior study that found an adverse effect of GV on cardiovascular complications and mortality risk among people without diabetes like that of among patients with diabetes^11^, we expect that greater GV may further deteriorate the risk of incident depression among healthy people without diabetes. Thus, given that depression is one of the leading causes of disability and imposes a remarkable burden worldwide, understanding the factors that increase the risk of its incidence is a major public health interest^10,15^. While depression has long been recognized as such a common complication of diabetes^16^, it is more prevalent and severe in patients with greater glycemic instability^7,10,17^. Therefore, we aimed to investigate the association between variability in visit-to-visit fasting serum glucose (FSG) levels and the incidence of depression in healthy Asian populations without diabetes using a nationally representative cohort from South Korea.

## Methods

### Study population

We extracted data from the Korean National Health Insurance Service-National Health Screening Cohort (NHIS-HEALS) database between January 1, 2002, and December 31, 2013. The NHIS has offered health insurance for all Korean citizens since 1989^18^ and provided biennial health screening examinations for all citizens aged ≥ 40 years old. During the examination, participants undergo urine and blood tests and basic physical measurements including height, weight, and blood pressure. Additionally, participants are required to fill out self-reported questionnaires regarding their health behavior, such as smoking, drinking, physical activity, and personal and family history of diseases. These collected data is then combined with sociodemographic information, hospital usage, and death register information, which would made into a subset dataset, called the NHIS-HEALS by a simple random sampling method. The NHIS-HEALS dataset offers a unique opportunity to explore the Korean population’s health-related characteristics^19^.

**Figure 1:**
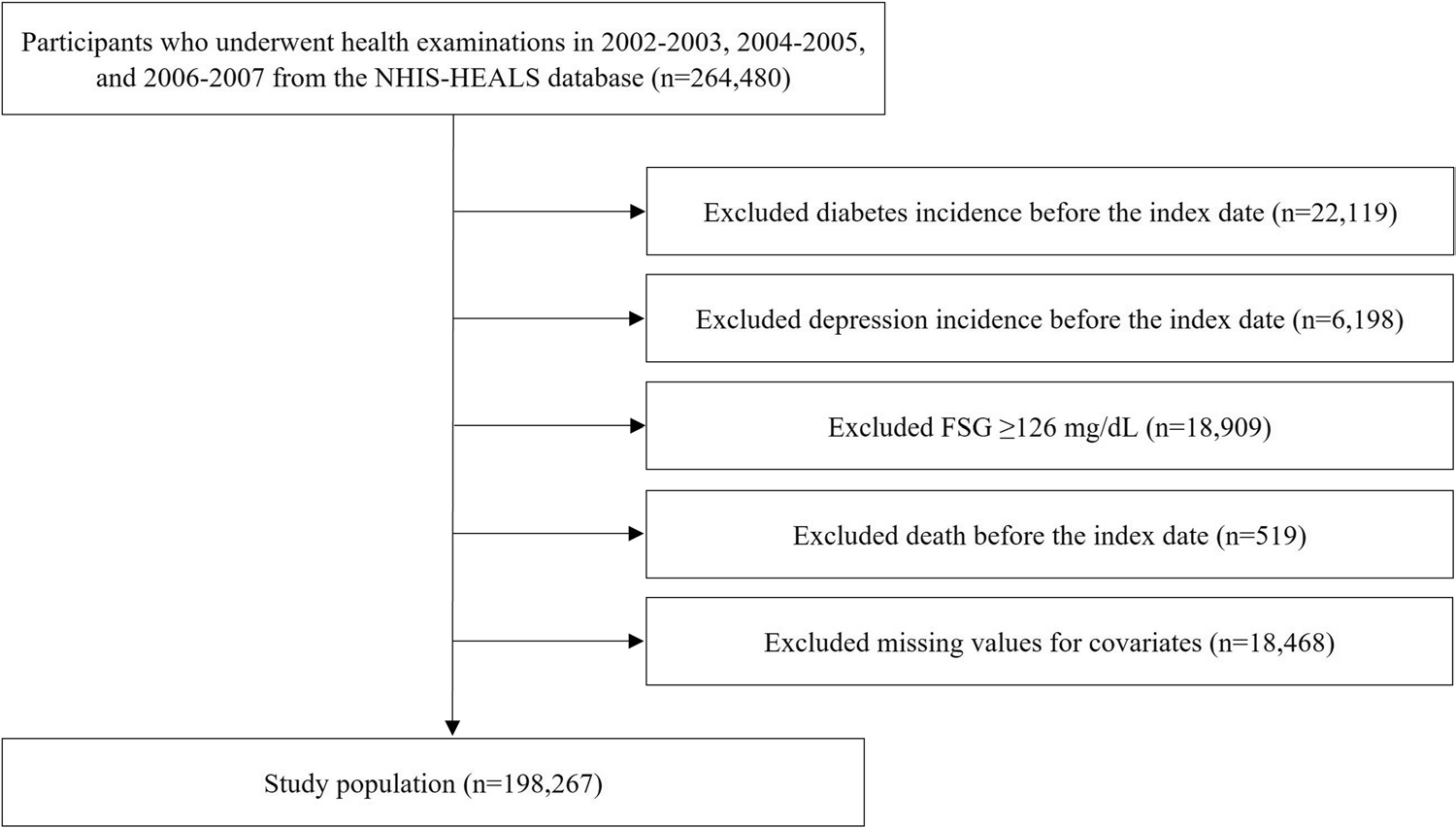
Flow diagram for the inclusion of study population. NHIS-HEALS, Korean National Health Insurance Service-National Health Screening Cohort; FSG, fasting serum glucose.

In total, 264,480 participants who underwent at least three health screening visits among the first (2002–2003), second (2004–2005), and third (2006–2007) health examinations and had FSG values were selected during screening. Among these, we excluded 22,119 participants diagnosed with diabetes and 6198 participants diagnosed with depression before the index date (January 1, 2008); 18,909 participants whose baseline FSG levels were ≥ 126.0 mg/dL in any of their health examinations; 519 participants who died before the index date; and 18,468 participants for missing data on covariates. Finally, our study population comprised 198,267 participants. The flowchart of our study is shown in Fig. 1.

### Study variables

GV was calculated as the average successive variability (ASV) of the FSG values^9^. Specifically, GV was driven by calculating the average absolute values of the differences in FSG between the successive examinations^20^. The study population was then distributed into five groups according to the extent of GV. The first quintile represented the lowest GV, whereas the fifth quintile represented the highest GV. Depression was defined according to codes from the 10th revision of the International Statistical Classification of Diseases and Related Health Problems (ICD-10) pertaining to depressive symptoms during the follow-up duration. ICD-10 is a standardized code for medical conditions and procedures established by the World Health Organization^21^. We considered incident depression as diagnosed with F32 (all mild to severe depressive episodes) or F33 (all recurrent depressive disorders) based on the ICD-10 code, along with the prescription of antidepressants.

### Statistical analysis

The clinical course of the participants was followed up from January 1, 2008, until the date of depression diagnoses, death, or December 31, 2013, whichever came first. Moreover, we censored any patients diagnosed with diabetes during the follow-up period, mainly to preclude diabetes as a risk factor for depression and to solely determine the effect of GV as a predictor of depression. The risk of depression due to GV was determined by calculating the hazard ratios (HR) and 95% confidence intervals (CIs) using Cox proportional hazards regression analysis. In all our analyses, the first quintile of GV, which indicated the lowest variability, was used as the reference group. We then calculated *p* for trend values to detect any trend between GV and depression risk.

We adjusted for the following potential confounding covariates: age (continuous; years), sex (categorical; male or female), baseline FSG level (continuous; mg/dL), change in FSG level (continuous; mg/dL), household income (categorical; first, second, third, and fourth quartiles), body mass index (continuous; kg/m^2^), smoking (categorical; never, past, and current), alcohol consumption (categorical; 0, < 1, 1–2, 3–4, and ≥ 5 times per week), physical activity (categorical; 0, 1–2, 3–4, 5–6, and ≥ 7 times per week), blood pressure (continuous; mmHg), total cholesterol level (continuous; mg/dL), and the Charlson comorbidity index (continuous). Change in FSG, which meant to consider the direction of glucose change, was calculated as the difference in FSG values between the third and first health examinations. We used insurance premium as the proxy for household income and calculated the Charlson comorbidity index using the same algorithms as those reported in another study^22^.

Stratified analyses were conducted to further investigate any differences in the outcome according to subgroups. The subgroups included sex, impaired FSG status, direction of FSG change, household income, body mass index, smoking, physical activity, alcohol consumption, and the Charlson comorbidity index. Furthermore, sensitivity analyses were conducted after excluding the incidence of depression in the first 1–5 years from the index date. In addition, the coefficient of variation (CV) of the FSG values was calculated as an alternative metric to support the primary findings. CV was calculated by dividing the standard deviation of the FSG values by the mean values and is one of the most commonly used metrics for measuring the degree of variability^23^.

Data collection was conducted using SAS version 9.4 (SAS Institute, Cary, NC, USA), and statistical analyses were performed using STATA version 13.0 (StataCorp, College Station, TX, USA). We used the chi-squared test to analyze categorical variables and analysis of variance for continuous variables to compare baseline characteristics according to the GV. Statistical significance was defined as a two-sided* p* value < 0.05.

### Ethical considerations

This study was approved by the Seoul National University Hospital Institutional Review Board (IRB number, X-1701/378–902). The requirement for informed consent from the participants was waived by the Institutional Review Board of Seoul National University Hospital on account of the retrospective nature of this study, and the NHIS-HEALS database was anonymized according to strict confidentiality guidelines prior to distribution. All the study methods were carried out in accordance with relevant guidelines and regulations.

## Results

A total of 264,480 people underwent the first (2002–2003), second (2004–2005), and third (2006–2007) health checkups, and 198,267 of them underwent follow-up for 6 years. We excluded patients who were diagnosed with diabetes (n = 22,119) and depression (n = 6198) before the index date. Participants with an FSG level ≥ 126 mg/dL (n = 18,909), who died before the index date (n = 519), and those with missing covariates (n = 18,468) during the study period were excluded.

Table 1 depicts the baseline characteristics of the study population according to GV. Among the total included participants, 20.95%, 20.45%, 20.68%, 18.13%, and 19.79% of participants were grouped into the first, second, third, fourth, and fifth quintiles of GV, respectively. The mean values of GV for each quintile are 3.35, 6.75, 9.92, 13.79, and 22.21 mg/dL, respectively. Compared with people with the lowest GV, those with the highest GV were more likely to be older, male, have lower household income, be current smokers, consume alcohol, engage in lower physical activity, and have more comorbidities.

**Table 1:** Descriptive characteristics of the study population.

	Glycemic Variability (GV)					*p* value
	First quintile	Second quintile	Third quintile	Fourth quintile	Fifth quintile	
**Number of people**	41,537	40,548	41,002	35,942	39,238	
**Age, years, mean (SD)**	55.2 (8.5)	55.4 (8.5)	55.6 (8.6)	55.9 (8.8)	56.5 (9.0)	< 0.001
**Sex, n (%)**						
Males	53.4	54.3	56.2	58.7	61.3	< 0.001
Females	46.6	45.7	43.8	41.3	38.7	
**GV (ASV), mg/dL mean (SD)**	3.3 (1.3)	6.8 (0.9)	9.9 (1.0)	13.8 (1.3)	22.2 (5.5)	< 0.001
Initial FSG, mg/dL, mean (SD)	90.0 (9.6)	89.9 (10.6)	89.8 (11.8)	90.2 (13.2)	91.2 (15.3)	< 0.001
Change in FSG, mg/dL, mean (SD)	0.3 (5.7)	1.1 (10.4)	2.3 (14.4)	3.1 (18.1)	3.0 (20.6)	< 0.001
**Household income, n (%)**						
First quartile (highest)	43.9	42.4	40.2	38.2	34.1	< 0.001
Second quartile	26.7	27.5	28.4	28.7	30.4	
Third quartile	18.1	18.7	19.3	20.1	21.5	
Fourth quartile (lowest)	11.2	11.5	12.2	13.0	13.9	
**Body mass index, kg/**${\mathbf{m}}^{2}$** mean (SD)**	23.8 (2.8)	23.8 (2.8)	23.9 (2.8)	23.9 (2.8)	23.9 (2.9)	< 0.001
**Smoking, n (%)**						
Never smoker	74.1	73.7	72.3	71.1	69.6	< 0.001
Past smoker	9.3	9.3	9.4	9.7	9.0	
Current smoker	16.6	17.0	18.2	19.2	21.4	
**Alcohol consumption, times per week, n (%)**						
None	60.0	59.6	58.5	57.7	56.5	< 0.001
< 1	15.6	15.8	15.5	15.2	14.8	
1–2	17.1	16.6	17.4	17.5	18.0	
3–4	5.1	5.5	5.8	6.5	6.8	
≥ 5	2.3	2.6	2.8	3.2	4.0	
**Physical activity, times per week, n (%)**						
None	45.5	46.2	46.9	48.4	50.2	< 0.001
1–2	30.0	29.5	29.9	28.7	27.9	
3–4	14.6	14.2	13.6	13.2	12.3	
5–6	3.6	3.5	3.3	3.5	3.1	
7	6.3	6.6	6.3	6.3	6.6	
**Systolic blood pressure, mmHg, mean (SD)**	124.1 (15.7)	124.3 (15.6)	124.8 (15.8)	125.6 (16.0)	126.3 (16.1)	< 0.001
**Total cholesterol, mg/dL, mean (SD)**	197.8 (35.1)	198.4 (35.4)	198.7 (35.8)	199.0 (36.3)	199.5 (37.0)	< 0.001
**Charlson comorbidity index, %**						
0	24.5	24.0	24.1	23.6	22.6	< 0.001
1–2	48.4	48.3	47.6	47.1	46.6	
≥ 3	27.2	27.7	28.3	29.4	30.8	

The *p* value was calculated by the chi-squared test for categorical variables and analysis of variance for continuous variables.

*FSG* fasting serum glucose, *SD* standard deviation, *n* number of people.

**Figure 2:**
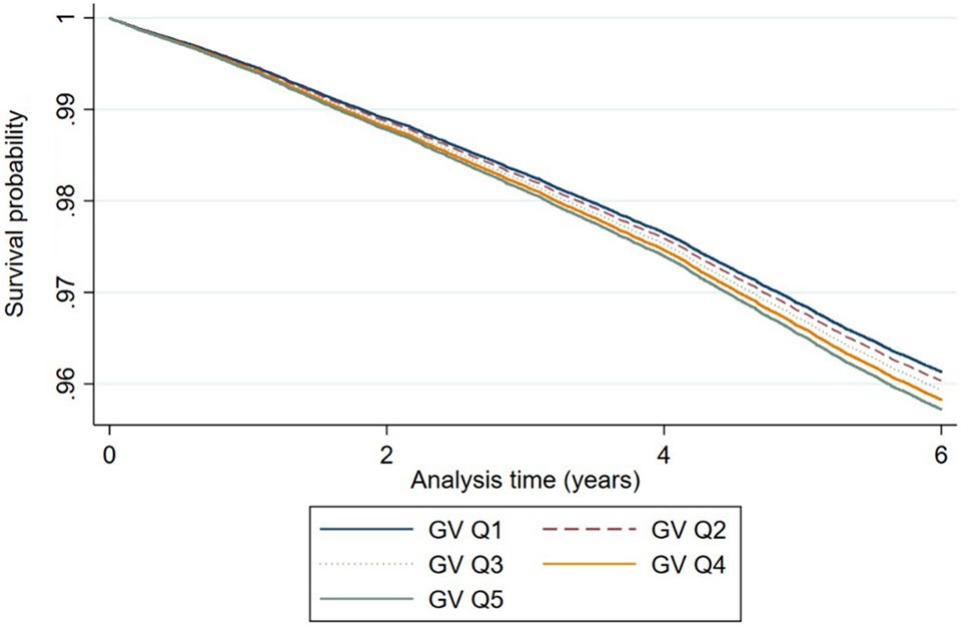
Kaplan–Meier estimation on association of glucose variability with risk of depression according to quintiles. GV, glucose variability; Q1, first quintile; Q2, second quintile; Q3, third quintile; Q4, fourth quintile; Q5, fifth quintile.

During the 6-year follow-up, 9244 participants developed depression. The results of the main analyses are shown in Table 2. People in the fifth quintile (highest) of GV had a significantly higher risk of incident depression than those in the first quintile (lowest) of GV (HR, 1.09; 95% CI, 1.02–1.16). Furthermore, the risk of developing depression increased upon greater extent of GV (*p* for trend < 0.001). All results were significant after adjusting for the covariates. Furthermore, we analyzed Kaplan–Meier curves according to GV quintiles. Likewise, the Kaplan–Meier cumulative risks for depression were higher in the fifth quintile group than the first quintile group of GV. The result is included in Fig. 2.

**Table 2:** Hazard ratios for depression according to GV.

All population	Glycemic variability (GV)					*p* for trend
	First quintile	Second quintile	Third quintile	Fourth quintile	Fifth quintile	
Events	1853	1812	1900	1748	1931	< 0.001
Person-years	238,203	231,842	233,293	203,491	220,184	
HR (95% CI)	1.00 (reference)	1.00 (0.94–1.07)	1.04 (0.97–1.11)	1.09 (1.02–1.16)	1.09 (1.02–1.16)	

The hazard ratio was calculated by Cox proportional hazards regression analysis after adjusting for age, sex, initial FSG, change in FSG, household income, body mass index, smoking, alcohol consumption, physical activity, systolic blood pressure, total cholesterol, and the Charlson comorbidity index.

*FSG* fasting serum glucose, *HR* hazard ratio, *CI* confidence interval.

We further conducted stratified analyses according to subgroups of sex, impaired FSG status, direction of change in FSG, household income, body mass index, smoking, physical activity, alcohol consumption, and the Charlson comorbidity index. For the subgroups of sex, we re-calculated the GV and its quintiles according to sex on account of slight unbalanced sex ratio of the study population (56.69% male and 42.31% female). No significant interaction was noted between GV and the onset of depression for any of these variables. The risk of developing depression appeared to be significantly higher upon greater GV in both sexes, people with normal FSG level (< 100 mg/dL), with any direction of FSG change, people within upper half household income level, any level of body mass index, people who never smoke, who ever do physical activities per week, who never drink, and whom with any comorbidities. The results are summarized in Table 3.

**Table 3:** Stratified analysis of the effect of GV on depression.

	Glycemic variability (GV)					*p* for trend	*p* for interaction
	First quintile	Second quintile	Third quintile	Fourth quintile	Fifth quintile		
**Sex**							
**Males, events**	751	714	704	680	773	0.023	0.625
Person-years	148,398	126,167	127,480	113,132	125,647		
HR (95% CI)	1.00	1.10	1.06	1.14	1.12		
	(reference)	(1.00–1.22)	(0.96–1.18)	(1.03–1.27)	(1.01–1.24)		
**Females, events**	1211	940	1222	1070	1179	0.035	
Person-years	110,518	88,465	106,366	89,377	91,464		
HR (95% CI)	1.00	0.96	1.02	1.04	1.07		
	(reference)	(0.88–1.04)	(0.94–1.10)	(0.96–1.13)	(0.98–1.16)		
**Impaired FSG (FSG ≥ 100** **mg/dL)**							
**No, events**	1455	1190	1109	698	389	0.006	0.447
Person-years	184,808	156,475	130,666	82,492	43,430		
HR (95% CI)	1.00	0.96	1.07	1.07	1.12		
	(reference)	(0.89–1.04)	(0.99–1.16)	(0.97–1.17)	(1.00–1.26)		
**Yes, events**	398	622	791	1050	1542	0.091	
Person-years	53,395	75,368	102,628	120,999	176,754		
HR (95% CI)	1.00	1.08	1.00	1.11	1.09		
	(reference)	(0.96–1.23)	(0.89–1.13)	(0.99–1.25)	(0.98–1.22)		
**Direction of change in FSG (3rd–1st)**							
**Increase, events**	1027	1025	1114	999	1105	0.007	0.994
Person-years	131,129	129,671	134,270	118,348	124,951		
HR (95% CI)	1.00	1.01	1.05	1.08	1.11		
	(reference)	(0.93–1.10)	(0.97–1.15)	(0.99–1.18)	(1.01–1.20)		
**Decrease, events**	826	787	786	749	826	0.029	
Person-years	107,074	102,171	99,023	85,143	95,234		
HR (95% CI)	1.00	0.99	1.02	1.10	1.08		
	(reference)	(0.89–1.09)	(0.93–1.13)	(1.00–1.22)	(0.97–1.19)		
**Household income**							
**Upper half (higher), events**	1220	1191	1221	1102	1180	0.001	0.488
Person-years	168,844	162,532	160,499	136,881	142,764		
HR (95% CI)	1.00	1.01	1.05	1.10	1.11		
	(reference)	(0.93–1.09)	(0.97–1.13)	(1.02–1.20)	(1.03–1.21)		
**Lower half(lower), events**	633	621	679	646	751	0.102	
Person-years	69,359	69,310	72,794	66,610	77,421		
HR (95% CI)	1.00	0.98	1.03	1.07	1.06		
	(reference)	(0.88–1.10)	(0.92–1.15)	(0.96–1.19)	(0.96–1.18)		
**Body mass index**							
** < 25, events**	1313	1244	1298	1174	1296	0.016	0.427
Person-years	165,049	159,898	158,348	137,074	146,508		
HR (95% CI)	1.00	0.97	1.02	1.06	1.07		
	(reference)	(0.90–1.05)	(0.95–1.10)	(0.98–1.15)	(0.99–1.15)		
** ≥ 25, events**	540	568	602	574	635	0.007	
Person-years	73,154	71,945	74,945	66,417	73,676		
HR (95% CI)	1.00	1.06	1.09	1.16	1.15		
	(reference)	(0.95–1.20)	(0.97–1.22)	(1.03–1.31)	(1.02–1.29)		
**Smoking**							
**Non-smoker, events**	1547	1478	1561	1411	1549	< 0.001	0.362
Person-years	176,227	170,650	168,588	144,567	152,830		
HR (95% CI)	1.00	0.98	1.04	1.09	1.10		
	(reference)	(0.91–1.05)	(0.97–1.12)	(1.01–1.17)	(1.03–1.18)		
**Ever-smoker, events**	306	334	339	337	382	0.586	
Person-years	61,976	61,192	64,706	58,924	67,354		
HR (95% CI)	1.00	1.09	1.03	1.09	1.05		
	(reference)	(0.93–1.27)	(0.88–1.20)	(0.94–1.28)	(0.90–1.22)		
**Physical activity (per week)**							
**No, events**	984	982	1022	953	1121	0.069	0.267
Person-years	107,613	106,426	108,726	97,532	109,637		
HR (95% CI)	1.00	1.00	1.02	1.05	1.07		
	(reference)	(0.92–1.10)	(0.93–1.11)	(0.96–1.15)	(0.98–1.17)		
**Yes, events**	869	830	878	795	810	0.001	
Person-years	130,590	125,416	124,567	105,959	110,548		
HR (95% CI)	1.00	0.99	1.06	1.14	1.12		
	(reference)	(0.90–1.09)	(0.97–1.17)	(1.04–1.26)	(1.01–1.23)		
**Drinking (per week)**							
**No, events**	1343	1281	1356	1212	1348	0.002	0.810
Person-years	142,077	137,214	135,673	116,552	123,231		
HR (95% CI)	1.00	0.98	1.04	1.08	1.10		
	(reference)	(0.91–1.06)	(0.97–1.12)	(1.00–1.16)	(1.01–1.18)		
**Yes, events**	510	531	544	536	583	0.098	
Person-years	96,126	94,628	97,621	86,939	96,953		
HR (95% CI)	1.00	1.05	1.03	1.12	1.09		
	(reference)	(0.93–1.19)	(0.91–1.16)	(0.99–1.27)	(0.96–1.22)		
**Charlson comorbidity index**							
**No comorbidity, events**	201	202	221	152	177	0.974	0.144
Person-years	59,604	57,106	57,622	49,396	51,579		
HR (95% CI)	1.00	1.05	1.15	0.93	1.05		
	(reference)	(0.86–1.28)	(0.95–1.40)	(0.75–1.15)	(0.86–1.29)		
**Yes comorbidity, events**	1652	1610	1679	1596	1754	< 0.001	
Person-years	178,599	174,736	175,671	154,095	168,606		
HR (95% CI)	1.00	0.99	1.03	1.12	1.11		
	(reference)	(0.93–1.06)	(0.96–1.10)	(1.04–1.20)	(1.03–1.19)		

Stratified analysis of the effect of GV on depression according to subgroups of sex, impaired FSG, direction of change in FSG, household income, body mass index, smoking, physical activity, drinking, and the Charlson comorbidity index. The hazard ratio was calculated by Cox proportional hazards regression analysis after adjustments for age, sex, initial FSG, change in FSG, household income, body mass index, smoking, alcohol consumption, physical activity, systolic blood pressure, total cholesterol, and the Charlson comorbidity index.

*FSG* fasting serum glucose, *HR* hazard ratio, *CI* confidence interval.

**Figure 3:**
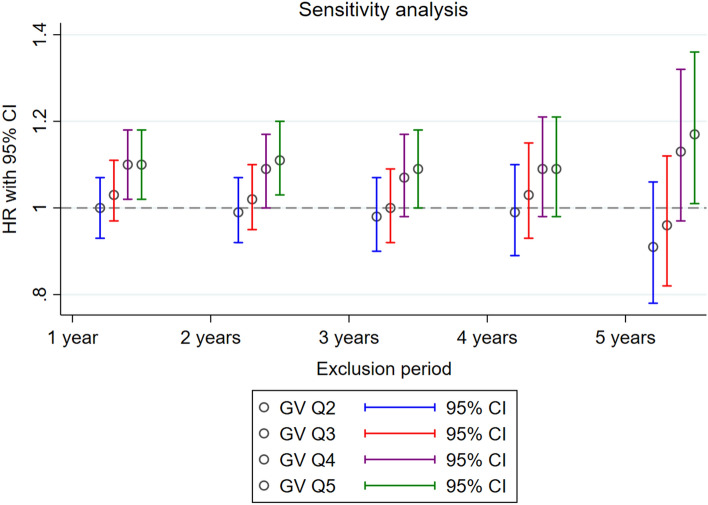
Summarized hazard ratios of sensitivity analysis. Sensitivity analysis of the effect of GV on depression after excluding participants with events occurring within the first 1–5 years of follow-up. The hazard ratio calculated by Cox proportional hazards regression analysis after adjustments for age, sex, initial FSG, change in FSG, household income, body mass index, smoking, alcohol consumption, physical activity, systolic blood pressure, total cholesterol, and the Charlson comorbidity index. HR, hazard ratio; CI, confidence interval; GV, glucose variability; Q2, second quintile; Q3, third quintile; Q4, fourth quintile; Q5, fifth quintile.

Furthermore, sensitivity analyses were conducted to evaluate the robustness of the results. First, we excluded the incidence of depression within 1–5 years of follow-up to solely examine the effect of GV as a potential risk factor for depression, and not as a prognostic symptom of its incidence. The results were consistent with the main results in which people with the highest GV showed a higher risk of incident depression. Moreover, the risk of developing depression appeared to be significantly higher upon greater GV (*p* for trend < 0.05) in all analyses. The results are summarized in Table 4 and Fig. 3. Second, the exact same Cox proportional hazards regression analyses were carried out using CV to define the GV. People in the fifth quintile (highest) of GV had a significantly higher risk of incident depression than those in the first quintile (lowest) of GV (HR, 1.10; 95% CI, 1.03–1.17), which is consistent with our findings based on ASV to define GV. Furthermore, the risk of developing depression increased with greater GV extent (*p* for trend = 0.004). Additionally, the results remained consistent even after excluding the incidence of depression within 1–5 years of follow-up. The results of using an alternative measure of variability are shown in Supplementary Tables S1 and S2.

**Table 4:** Sensitivity analysis of the effect of GV on depression.

Exclusion period	Glycemic variability (GV) HR (95% CI)					*p* for trend
	First quintile	Second quintile	Third quintile	Fourth quintile	Fifth quintile	
One year	1.00 (reference)	1.00 (0.93–1.07)	1.03 (0.97–1.11)	1.10 (1.02–1.18)	1.10 (1.02–1.18)	0.001
Two years	1.00 (reference)	0.99 (0.92–1.07)	1.02 (0.95–1.10)	1.09 (1.00–1.17)	1.11 (1.03–1.20)	0.001
Three years	1.00 (reference)	0.98 (0.90–1.07)	1.00 (0.92–1.09)	1.07 (0.98–1.17)	1.09 (1.00–1.18)	0.012
Four years	1.00 (reference)	0.99 (0.89–1.10)	1.03 (0.93–1.15)	1.09 (0.98–1.21)	1.09 (0.98–1.21)	0.033
Five years	1.00 (reference)	0.91 (0.78–1.06)	0.96 (0.82–1.12)	1.13 (0.97–1.32)	1.17 (1.01–1.36)	0.002

Sensitivity analysis of the effect of GV on depression after excluding participants with events occurring within the first 1–5 years of follow-up. The hazard ratio calculated by Cox proportional hazards regression analysis after adjustments for age, sex, initial FSG, change in FSG, household income, body mass index, smoking, alcohol consumption, physical activity, systolic blood pressure, total cholesterol, and the Charlson comorbidity index.

*FSG* fasting serum glucose, *HR* hazard ratio, *CI* confidence interval.

## Discussion

In this retrospective cohort study, we examined the association between visit-to-visit variability of FSG and the risk of depression among 198,267 Koreans without diabetes. The results revealed that GV is a significant predictor of the incidence of depression in people without diabetes. Among individuals with a greater GV, the risk of developing depression was significantly higher than those with the lowest GV after adjusting for confounding factors. To the best of our knowledge, this is the first study to report the greater GV as a risk factor for incident depression among people without diabetes.

According to earlier studies, people with normal glucose tolerance can also experience some degree of GV, and such oscillating glucose has greater detrimental effects on endothelial function and oxidative stress than constant high glucose in both people with and without diabetes^13^. An elevated risk of depression is linked to these biological changes. Along with the fact that GV significantly increases with age in the general adult population, evaluating the effect of GV in people without diabetes is crucial^11^. In addition, understanding the distribution and impact of GV in general populations is important for interpreting reference values. Given the goal of diabetes treatment is to restore glycemia to that of people without diabetes, the investigation of potential harm following GV should begin with examinations of those without diabetes, allowing for the determination of the point at which GV becomes pathologically significant^14^. Therefore, GV may also be a risk factor that should be monitored in a normal population without diabetes.

Consistent with our results, several studies have confirmed the association between visit-to-visit GV and depression, although their samples consisted of people with diabetes and relied on other glucose parameters rather than FSG^7,10^. For example, a recent study which investigated the effect of fasting plasma glucose coefficient of variation (FPG-CV) on the development of depression found that patients with type 2 diabetes and the highest 1-year FPG-CV had an elevated risk of depression^10^. Another study revealed that an increased variability in HbA1c is associated with higher risk of complications such as depression, though the study population was limited to elderly patients with type 2 diabetes^7^.

Several mechanisms may possibly underly the association between GV and incidence of depression. Given that visit-to-visit GV correlates with the mean concentration of blood glucose or HbA1c, it reflects the hyperglycemia status to a certain extent. While similar mechanisms may engage in the development of depression upon greater GV^24^, GV induces greater oxidative stress^15,25,26^ and endothelial dysfunction^13,27^ compared to single abnormal glycemic level^11,28^. These two mechanisms are commonly discussed as pathways that activate the pathogenesis of abnormalities in the neuroendocrine^10,29,30^ caused by GV^13,31^. Specifically, oscillating glycemic induces reactive oxygen species (ROS), leading to the activation of protein kinase C^32,33^, which, in turn, triggers apoptosis in endothelial cells^32,34^. In addition, through a transcription factor called nuclear factor kappa B (NF-κB), GV may fuel the concentration of pro-inflammatory cytokines and expression of adhesion molecules in endothelial cells^32,33^, which promote the incident depression^31,35^. Furthermore, the enhanced oxidative stress is further related to greater telomere shortening according to in vitro models^36,37^, which is often observed in neuropsychiatric disorders, including depression^38^. However, because most studies have been conducted on people with diabetes or in in vitro settings, it is unknown whether the same mechanisms of action can be applied to people without diabetes^11^.

The following limitations must be considered when interpreting our results. First, the retrospective nature of our study makes it difficult to fully control for all the factors that may affect GV and depression. For example, factors regarding dietary, behavioral, and socioeconomic characteristics may incur GV or depression^39^. According to independent studies dealt with glycemic control and depression respectively, factors like high-carbohydrate diet^40^, low physical activity^41^, consumption of alcohol^42^, smoking^43^, or higher income level^41^ can increase FSG. Moreover, depression is more prevalent among people who smoke, lack physical activity, consume excessive alcohol^44^, or those who are obese^45,46^, with lower income level^47^, or with chronic diseases^45,47^. Though we adjusted for as many confounding factors as possible including smoking, physical activity, alcohol intake, body mass index, household income, and the Charlson comorbidity index, other factors may further engage in the association between GV and incident depression. Therefore, dedicated studies based on larger individual-level information would be needed as to elucidate the link between GV and incident depression among people without diabetes. Second, the population in our sample is restricted to those who have undergone at least three health examinations, are probably in a certain socioeconomic group, or are more concerned with their own health. Additionally, close to 28% of our sample had three or more comorbidities, indicating that they were not in good health. These may reduce the generalizability of this study, which would mean that the findings of this study might not be applicable to populations with better health^23^. The Charlson comorbidity index and household income adjustments were attempts to address this issue, but external validation is still required before we can generalize our findings. Third, association surveys continue to serve as the foundation for hypotheses that are tested through more rigorous prospective research, typically randomized clinical trials^48^. Therefore, despite the fact that our study's large sample size from a representative cohort of Koreans dwarfs that of earlier studies and offers compelling evidence for the significance of GV in predicting future depression risk, the possibility of reversibility between the two factors should be considered. Additional study is needed to elucidate the causal relationship between GV and depression risk. Last but not least, while we used ASV of FSG among other GV indices, several other ways can be used to assess GV. Though there is no gold standard for defining GV^11^, the association between GV and incident depression may vary according to the measure taken for calculating GV^1,49^. Further studies based on other GV measures are warranted to enhance the generalizability of our results.

Despite the aforementioned limitations, our study is with some contributions. To the best of our knowledge, this is the first large-scale study to discover the relationship between GV and the risk of developing depression in people without diabetes. Our study has crucial clinical implications for people without diabetes who have generally been neglected in the past. Given the findings of our study, individuals without diabetes may develop depression when they have greater variability in fasting glycemic levels. Therefore, carefully monitoring an individual’s visit-to-visit GV could contribute to the prevention and reduction of the risk of developing depression as well as diabetes. Furthermore, we could also emphasize the importance of maintaining a certain level of stability of depression risk factors, as other studies have reported increased depression risk following greater variability of its other metabolic risk factors. Short-term systolic blood pressure variability, for example, has been widely linked to an increased risk of depression onset^50^. Also, depressed people have lower heart rate variability values than healthy people, indicating that this parameter could be used as a diagnostic and predictive biomarker of depression^51^. Hence, this study highlights the need for additional research into the impact of variability in depression risk factors.

In conclusion, greater visit-to-visit GV may increase the risk of developing depression even in people without diabetes. The association held true after considering people’s physical, psychological, and environmental factors. Maintaining a steady FSG level should be recommended to lower the risk of incident depression as well as diabetes. Larger studies are warranted to verify the causality between GV and depression among people without diabetes.

## Supplementary Information

Supplementary Information.

## Data Availability

The data used in this study are available from the Korean NHIS through a formal online proposal at https://nhiss.nhis.or.kr.

## Abbreviations

GV: Glycemic variability
NHIS-HEALS: National Health Insurance Service–National Health Screening Cohort
FSG: Fasting serum glucose
ASV: Average successive variability
ICD-10: 10th revision of the International Statistical Classification of Diseases and Related Health Problems
HR: Hazard ratio
CI: Confidence interval
FPG-CV: Fasting plasma glucose coefficient of variation

## References

[1] Satya Krishna SV, Kota SK, Modi KD (2013). Glycemic variability: Clinical implications. Indian J. Endocrinol. Metab..

[2] Zhou Z, Sun B, Huang S, Zhu C, Bian M (2020). Glycemic variability: Adverse clinical outcomes and how to improve it?. Cardiovasc. Diabetol..

[3] Papachristoforou E, Lambadiari V, Maratou E, Makrilakis K (2020). Association of glycemic indices (hyperglycemia, glucose variability, and hypoglycemia) with oxidative stress and diabetic complications. J. Diabetes Res..

[4] Hirakawa Y (2014). Impact of visit-to-visit glycemic variability on the risks of macrovascular and microvascular events and all-cause mortality in type 2 diabetes: The ADVANCE trial. Diabetes Care.

[5] Bruginski D, Précoma DB, Sabbag A, Olandowski M (2020). Impact of glycemic variability and hypoglycemia on the mortality and length of hospital stay among elderly patients in Brazil. Curr. Diabetes Rev..

[6] Gorst C (2015). Long-term glycemic variability and risk of adverse outcomes: A systematic review and meta-analysis. Diabetes Care.

[7] Ravona-Springer R (2017). Hemoglobin A1c variability predicts symptoms of depression in elderly individuals with type 2 diabetes. Diabetes Care.

[8] Li T-C (2017). Visit-to-visit variations in fasting plasma glucose and HbA1c associated with an increased risk of Alzheimer disease: Taiwan Diabetes Study. Diabetes Care.

[9] Bancks MP (2018). Fasting glucose variability in young adulthood and cognitive function in middle age: The Coronary Artery Risk Development in Young Adults (CARDIA) Study. Diabetes Care.

[10] Li CI (2020). Competing risk analysis on visit-to-visit glucose variations and risk of depression: The Taiwan Diabetes Study. Diabetes Metab.

[11] Yu JH (2019). Effects of long-term glycemic variability on incident cardiovascular disease and mortality in subjects without diabetes: A nationwide population-based study. Medicine (Baltimore).

[12] Wang C (2012). Glucose fluctuations in subjects with normal glucose tolerance, impaired glucose regulation and newly diagnosed type 2 diabetes mellitus. Clin. Endocrinol. (Oxf.).

[13] Ceriello A (2008). Oscillating glucose is more deleterious to endothelial function and oxidative stress than mean glucose in normal and type 2 diabetic patients. Diabetes.

[14] Gude F (2017). Glycemic variability and its association with demographics and lifestyles in a general adult population. J. Diabetes Sci. Technol..

[15] Ceretta LB (2012). Increased prevalence of mood disorders and suicidal ideation in type 2 diabetic patients. Acta Diabetol.

[16] Anderson RJ, Freedland KE, Clouse RE, Lustman PJ (2001). The prevalence of comorbid depression in adults with diabetes: A meta-analysis. Diabetes Care.

[17] Penckofer S (2012). Does glycemic variability impact mood and quality of life?. Diabetes Technol. Ther..

[18] Cheol Seong S (2017). Data resource profile: The National Health Information Database of the National Health Insurance Service in South Korea. Int. J. Epidemiol..

[19] Seong SC (2017). Cohort profile: The National Health Insurance Service-National Health Screening Cohort (NHIS-HEALS) in Korea. BMJ Open.

[20] Echouffo-Tcheugui JB (2019). Visit-to-visit glycemic variability and risks of cardiovascular events and all-cause mortality: The ALLHAT study. Diabetes Care.

[21] Mccarthy M, B. K. *ICD-10 Compliance: Process Improvement and Maintenance for Long-Term Care*. 318 (HCPro a division of BLR, 2015).

[22] Sundararajan V (2004). New ICD-10 version of the Charlson comorbidity index predicted in-hospital mortality. J. Clin. Epidemiol..

[23] Zhao MJ, Prentice JC, Mohr DC, Conlin PR (2021). Association between hemoglobin A1c variability and hypoglycemia-related hospitalizations in veterans with diabetes mellitus. BMJ Open Diabetes Res. Care.

[24] Lachin JM (2017). Association of glycemic variability in type 1 diabetes with progression of microvascular outcomes in the diabetes control and complications trial. Diabetes Care.

[25] Ohara M (2016). Relationship between daily and day-to-day glycemic variability and increased oxidative stress in type 2 diabetes. Diabetes Res. Clin. Pract..

[26] Chang CM, Hsieh CJ, Huang JC, Huang IC (2012). Acute and chronic fluctuations in blood glucose levels can increase oxidative stress in type 2 diabetes mellitus. Acta Diabetol..

[27] Thomas AJ, Kalaria RN, O'Brien JT (2004). Depression and vascular disease: What is the relationship?. J. Affect. Disord.

[28] Folli F (2011). The role of oxidative stress in the pathogenesis of type 2 diabetes mellitus micro-and macrovascular complications: Avenues for a mechanistic-based therapeutic approach. Curr. Diabetes Rev..

[29] Esposito K (2002). Inflammatory cytokine concentrations are acutely increased by hyperglycemia in humans: Role of oxidative stress. Circulation.

[30] Lustman PJ, Clouse RE (2005). Depression in diabetic patients: The relationship between mood and glycemic control. J. Diabetes Complicat..

[31] Vavakova M, Durackova Z, Trebaticka J (2015). Markers of oxidative stress and neuroprogression in depression disorder. Oxid. Med. Cell. Longev..

[32] Quagliaro L (2005). Intermittent high glucose enhances ICAM-1, VCAM-1 and E-selectin expression in human umbilical vein endothelial cells in culture: The distinct role of protein kinase C and mitochondrial superoxide production. Atherosclerosis.

[33] Hirsch IB (2005). Glycemic variability: It's not just about A1C anymore!. Diabetes Technol. Ther..

[34] Risso A, Mercuri F, Quagliaro L, Damante G, Ceriello A (2001). Intermittent high glucose enhances apoptosis in human umbilical vein endothelial cells in culture. Am. J. Physiol. Endocrinol. Metab..

[35] Jeon SW, Kim YK (2016). Neuroinflammation and cytokine abnormality in major depression: Cause or consequence in that illness?. World J. Psychiatry.

[36] Kawanishi S, Oikawa S (2004). Mechanism of telomere shortening by oxidative stress. Ann. N. Y. Acad. Sci..

[37] von Zglinicki T (2000). Role of oxidative stress in telomere length regulation and replicative senescence. Ann. N. Y. Acad. Sci..

[38] Simon NM (2006). Telomere shortening and mood disorders: Preliminary support for a chronic stress model of accelerated aging. Biol. Psychiatry.

[39] Lin LY (2019). Dietary patterns in relation to components of dyslipidemia and fasting plasma glucose in adults with dyslipidemia and elevated fasting plasma glucose in Taiwan. Nutrients..

[40] Mukherjee S, Thakur G, Kumar BD, Mitra A, Chakraborty C (2009). Long-term effects of a carbohydrate-rich diet on fasting blood sugar, lipid profile, and serum insulin values in rural Bengalis. J. Diabetes.

[41] Walatara KN, Athiththan LV, Hettiaratchi UK, Perera PR (2016). Effect of demographic status and lifestyle habits on glycaemic levels in apparently healthy subjects: A cross-sectional study. J. Diabetes Res..

[42] Lim J, Lee JA, Cho HJ (2018). Association of alcohol drinking patterns with presence of impaired fasting glucose and diabetes mellitus among South Korean adults. J. Epidemiol..

[43] Sultana R (2019). Fasting serum glucose level in male cigarette smoker. Mymensingh Med. J..

[44] Emerson ND (2018). Behavioral risk factors for self-reported depression across the lifespan. Mental Health Prevent..

[45] Strine TW (2008). Depression and anxiety in the United States: Findings from the 2006 Behavioral Risk Factor Surveillance System. Psychiatr. Serv..

[46] Gigantesco, A., Ferrante, G., Baldissera, S., Masocco, M. & Group, P. C. Depressive symptoms and behavior-related risk factors, Italian population-based surveillance system, 2013. *Prev. Chronic Dis* **12**, E183. 10.5888/pcd12.150154 (2015). 10.5888/pcd12.150154 PMC4651141 26513439

[47] Turgunova L (2017). The incidence of depression among the population of Central Kazakhstan and its relationship with sociodemographic characteristics. Behav. Neurol..

[48] Krakoff LR, Phillips RA (2016). Blood pressure variability: insights from “Big Data”. J Am Coll Cardiol..

[49] Ajjan R, Slattery D, Wright E (2019). Continuous glucose monitoring: A brief review for primary care practitioners. Adv. Ther..

[50] Tully PJ, Debette S, Tzourio C (2018). The association between systolic blood pressure variability with depression, cognitive decline and white matter hyperintensities: The 3C Dijon MRI study. Psychol. Med..

[51] Brunoni AR (2013). Heart rate variability is a trait marker of major depressive disorder: Evidence from the sertraline vs. electric current therapy to treat depression clinical study. Int. J. Neuropsychopharmacol..

