# Supplementary material for: Association between visit-to-visit fasting glycemic variability and depression: a retrospective cohort study in a representative Korean population without diabetes
Source: Sci Rep. 2022 Nov 4;12:18692. doi: 10.1038/s41598-022-22302-0 (PMC9636237; doi:10.1038/s41598-022-22302-0)
Supplement: Supplementary file 1 — Supplementary Information. [file 41598_2022_22302_MOESM1_ESM.docx]

**Title**

Association between visit-to-visit fasting glycemic variability and depression: a retrospective cohort study in a representative Korean population without diabetes

**Authors**

Hye Jun Kim^1,2^, Sung Min Kim^3^, Gyeongsil Lee^4^, Seulggie Choi^3^, Joung Sik Son^4^, Yun Hwan Oh^5^, Soo Jung Choi^6^, Seogsong Jeong^1,3^, Sang Min Park^3,4^

**Author Affiliations**

^1^Department of Biomedical Informatics, CHA University School of Medicine, Seongnam, Korea (the Republic of)

^2^Department of Business Administration, Yonsei University Graduate School, Seoul, Korea (the Republic of)

^3^Department of Biomedical Sciences, Seoul National University Graduate School, Seoul, Korea (the Republic of)

^4^Department of Family Medicine, Seoul National University Hospital, Seoul, Korea (the Republic of)

^5^Department of Family medicine, Chung-Ang University Gwangmyeong Hospital, Chung-Ang University College of Medicine, Gwangmyeong-si, Korea (the Republic of)

^6^Department of Family Medicine, Gachon University Gil Medical Center, Incheon, Korea (the Republic of)

**Corresponding author:** Sang Min Park

Department of Family Medicine and Biomedical Sciences, College of Medicine, Seoul National University, 101 Daehak-ro, Jongno-gu, Seoul 03080, Korea (the Republic of)

Tel.: +82-2-2072-3331

Fax: +82-2-766-3276

E-mail: smpark.snuh@gmail.com

**Supplementary Table S1.** Hazard ratios for depression according to GV defined as CV

|  | **Glycemic Variability (GV)** | | | | | ***p* for trend** |
| --- | --- | --- | --- | --- | --- | --- |
| All population | **First quintile** | **Second quintile** | **Third quintile** | **Fourth quintile** | **Fifth quintile** |  |
| Events | 1,765 | 1,774 | 1,896 | 1,854 | 1,954 |  |
| Person-years | 227,260 | 226,184 | 225,604 | 225,075 | 222,891 |  |
| HR (95% CI) | 1.00 | 1.01 | 1.07 | 1.04 | 1.10 | 0.004 |
|  | (reference) | (0.95-1.08) | (1.00-1.14) | (0.98-1.11) | (1.03-1.17) |  |

The hazard ratio was calculated by Cox proportional hazards regression analysis after adjusting for age, sex, initial FSG, change in FSG, household income, body mass index, smoking, alcohol consumption, physical activity, systolic blood pressure, total cholesterol, and the Charlson comorbidity index.

CV, coefficient of variation; FSG, fasting serum glucose; HR, hazard ratio; CI, confidence interval

**Supplementary Table S2.** Sensitivity analysis of the effect of GV defined as CV on depression

|  | **Glycemic Variability (GV)**  **HR (95% CI)** | | | | | ***p* for trend** |
| --- | --- | --- | --- | --- | --- | --- |
| **Exclusion period** | **First quintile** | **Second quintile** | **Third quintile** | **Fourth quintile** | **Fifth quintile** |  |
| One year | 1.00 | 1.00 | 1.08 | 1.03 | 1.11 | 0.003 |
|  | (reference) | (0.93-1.08) | (1.01-1.16) | (0.96-1.11) | (1.04-1.19) |  |
| Two years | 1.00 | 0.98 | 1.08 | 1.01 | 1.11 | 0.004 |
|  | (reference) | (0.90-1.06) | (1.00-1.17) | (0.94-1.09) | (1.03-1.20) |  |
| Three years | 1.00 | 0.97 | 1.05 | 1.00 | 1.11 | 0.016 |
|  | (reference) | (0.88-1.06) | (0.96-1.15) | (0.92-1.10) | (1.01-1.21) |  |
| Four years | 1.00 | 1.03 | 1.09 | 1.05 | 1.16 | 0.007 |
|  | (reference) | (0.92-1.14) | (0.98-1.21) | (0.95-1.17) | (1.04-1.29) |  |
| Five years | 1.00 | 0.91 | 0.96 | 1.04 | 1.23 | 0.001 |
|  | (reference) | (0.78-1.07) | (0.82-1.12) | (0.90-1.22) | (1.06-1.43) |  |

Sensitivity analysis of the effect of GV on depression after excluding participants with events occurring within the first 1-5 years of follow-up. The hazard ratio calculated by Cox proportional hazards regression analysis after adjustments for age, sex, initial FSG, change in FSG, household income, body mass index, smoking, alcohol consumption, physical activity, systolic blood pressure, total cholesterol, and the Charlson comorbidity index.

CV, coefficient of variation; FSG, fasting serum glucose; HR, hazard ratio; CI, confidence interval
